# Advances in Protein-Ligand Binding Affinity Prediction *via* Deep Learning: A Comprehensive Study of Datasets, Data Preprocessing Techniques, and Model Architectures

**DOI:** 10.2174/0113894501330963240905083020

**Published:** 2024-09-24

**Authors:** Gelany Aly Abdelkader, Jeong-Dong Kim

**Affiliations:** 1 Department of Computer Science and Electronic Engineering, Sun Moon University, Asan 31460, Republic of Korea;; 2 Division of Computer Science and Engineering, Sun Moon University, Asan 31460, Republic of Korea;; 3 Genome-based BioIT Convergence Institute, Sun Moon University, Asan 31460, Korea

**Keywords:** Deep learning, protein-ligand binding affinity, compound-protein interaction, drug discovery, drug repurposing, DNA sequences

## Abstract

**Background:**

Drug discovery is a complex and expensive procedure involving several timely and costly phases through which new potential pharmaceutical compounds must pass to get approved. One of these critical steps is the identification and optimization of lead compounds, which has been made more accessible by the introduction of computational methods, including deep learning (DL) techniques. Diverse DL model architectures have been put forward to learn the vast landscape of interaction between proteins and ligands and predict their affinity, helping in the identification of lead compounds.

**Objective:**

This survey fills a gap in previous research by comprehensively analyzing the most commonly used datasets and discussing their quality and limitations. It also offers a comprehensive classification of the most recent DL methods in the context of protein-ligand binding affinity prediction (BAP), providing a fresh perspective on this evolving field.

**Methods:**

We thoroughly examine commonly used datasets for BAP and their inherent characteristics. Our exploration extends to various preprocessing steps and DL techniques, including graph neural networks, convolutional neural networks, and transformers, which are found in the literature. We conducted extensive literature research to ensure that the most recent deep learning approaches for BAP were included by the time of writing this manuscript.

**Results:**

The systematic approach used for the present study highlighted inherent challenges to BAP *via* DL, such as data quality, model interpretability, and explainability, and proposed considerations for future research directions. We present valuable insights to accelerate the development of more effective and reliable DL models for BAP within the research community.

**Conclusion:**

The present study can considerably enhance future research on predicting affinity between protein and ligand molecules, hence further improving the overall drug development process.

## 1 INTRODUCTION

The concept of drug repurposing has emerged as a promising strategy in modern drug discovery, offering a shortcut to identifying new therapeutic uses for existing drugs. Computational methods enable systematic exploration of the vast landscape of molecular interactions, facilitating the repurposing of approved drugs for novel indications [1]. This approach circumvents many of the challenges associated with traditional drug discovery and accelerates the identification of lead compounds with therapeutic potential.

Exploring compound-protein interactions (CPIs) is fundamental to drug discovery and repurposing, as they elucidate the mechanisms of action and therapeutic efficacy of pharmaceutical agents. DL methods play an essential role in elucidating these interactions, covering various aspects such as binding site prediction, molecular docking, binding interaction prediction framed as binary classification, and binding affinity prediction.

With binding site prediction algorithms, one can identify the regions on target proteins where small molecules (ligands) are likely to bind, providing valuable insights into potential drug-target interactions [2, 3]. On the other hand, molecular docking algorithms further facilitate the exploration of CPI by simulating the binding process and predicting the optimal orientation and conformation of ligands within binding sites [4]. BAP algorithms play a crucial role in quantifying the strength of interactions between compounds and target proteins, enabling the prioritization of lead compounds based on their potential efficacy. Additionally, binding interaction prediction, framed as binary classification, allows researchers to distinguish between active and inactive compounds based on their propensity to interact with target proteins [5, 6].

The availability of experimentally measured data on binding interactions has spurred the proliferation of numerous DL methods. The authors across the field propose diverse feature representations and architectures for feature learning, ranging from convolutional neural networks (CNNs) models to recurrent neural networks (RNNs), graph neural networks (GNNs), and even transformers. The diversity reflects the adaptability of DL methodologies in comprehensively capturing the complexities inherent in CPI.

CPI prediction has often been framed as a binary classification. Although this simplification has aided in developing predictive models, it has limitations. Binary classification tends to oversimplify the diverse nature of binding interactions, neglecting the varying degrees of affinity between compounds and proteins. Instead of recognizing a continuum of affinities, where interactions can range from weak to strong, binary classification tends to focus on a rigid categorization of interactions as binding or nonbinding. The approach overlooks the nuanced and varied strengths of molecular interactions inherent in biological systems.

In contrast, framing CPI prediction as a regression task to predict binding affinity offers a more informative perspective. Regression models provide valuable insights into the potency and efficacy of potential drug candidates by quantifying the strength of interactions between compounds and proteins. This nuanced understanding is crucial for prioritizing lead compounds and effectively guiding drug discovery. Therefore, our focus is specifically on studies that employ binding affinity as the target variable for predictive modelling in CPI.

### 1.1 Motivation

Numerous reviews have explored various aspects of protein-ligand binding affinity prediction, yet a notable gap persists in the examination of DL methodologies. For instance, Zhang *et al.* [7] concentrated solely on graph neural networks for BAP, while Bagherian *et al.* [8] focused their study on machine learning (ML) methods. Similarly, D. Wang *et al.* [9] focused on free energy-based simulations and ML-based scoring functions in their analysis. Although Wang *et al.* [10] delved into DL, they lacked comprehensive discussions on various data representations and commonly used datasets for BAP, with limited exploration of DL-based methods. Additionally, Meli *et al.* [11] predominantly focused on structure-based DL techniques, omitting the most up-to-date models.

Our aim is to bridge the gap by thoroughly examining commonly used datasets, discussing their quality, and offering an in-depth analysis of the most up-to-date DL methods, challenges, and future directions within this context. By addressing these crucial aspects, we aspire to provide a nuanced understanding of the role of DL in BAP, highlighting its potential implications for drug discovery and drug repurposing.

### 1.2 Contributions

The primary contributions of the study can be outlined as follows:

- In-depth data analysis and quality evaluation for informed dataset selection:

In contrast to previous surveys, we provide a richer data analysis and quality evaluation of the most used datasets in the BAP. By offering an in-depth assessment of dataset characteristics and quality, the paper guides the selection of adequate datasets for their studies.

- Comprehensive analysis of DL methods, with a focus on up-to-date methods:

Given the limited coverage of DL approaches in previous studies, we thoroughly analyzed various DL methods employed in BAP, notably by covering the most up-to-date DL methods from the literature and offering the most detailed classification compared to previous surveys. This comprehensive examination enhances our understanding of the efficacy of DL models in BAP.

- Identification of future directions for research and innovation:

We identify critical areas for further research and innovation by discussing the challenges and future directions in DL for BAP. By highlighting emerging trends and potential avenues for exploration, our survey guides researchers and practitioners toward advancing drug discovery and repurposing initiatives using state-of-the-art DL techniques.

As outlined in Fig. (**1**), the subsequent sections of this article are organized as follows. Section 2 provides a brief yet comprehensive background to foster a clear understanding of the topics at hand. In Section 3, we delve into an extensive data analysis and quality evaluation of the predominant datasets in the BAP. Section 4 discusses common data preprocessing methods, and Section 5 presents a comprehensive analysis of DL methods employed in BAP, detailing various methods and their limitations. In Section 6, we discuss challenges and directions for future works, highlighting critical areas for further exploration and innovation in DL for BAP.

## 2 BACKGROUND

The drug discovery process unfolds through several distinct stages, each marked by challenges and complexities. It typically begins with target identification and validation, wherein the molecular mechanisms underlying a disease or pathological condition are studied [12]. These potential targets encompass a diverse range of biomolecules, including DNA sequences, RNA molecules, proteins, and metabolites, which hold promise for therapeutic intervention. Once a target has been identified, the search for lead compounds commences, often through high-throughput screening of chemical libraries or rational drug design strategies.

After promising compound identification, a meticulous evaluation process ensues, subjecting each identified compound to rigorous scrutiny concerning its efficacy, safety profile, and pharmacological properties. Following this thorough evaluation, the selected compound advances to preclinical research, undergoing rigorous testing in both *in vitro* and *in vivo* settings.

This phase allows for a comprehensive assessment of the potential impact of compounds on physiological systems, laying the groundwork for subsequent clinical trials and regulatory approval processes. However, as outlined in Fig. (**2**), this traditional approach to drug discovery has some limitations. It is time-consuming, labor-intensive, and prohibitively costly, requiring extensive resources and years of painstaking effort [13, 14].

## 3 MOST USED DATA IN BAP

Recent advancements in high-throughput screening, open data initiatives, and sequencing technologies have enabled large-scale experiments for compound and gene characterization [15-20]. These experiments have led to the identification of novel CPI and contributed to the rapid growth of CPI databases. However, managing and curating these abundant data remains an ongoing challenge in computational biology [21]. For instance, it can be difficult to identify the most relevant and reliable CPI data for training a DL model. Additionally, the quality of CPI data can vary widely, making it challenging to evaluate their reliability.

Numerous databases and repositories for CPI data are available and can be categorized into protein-centric, compound-centric, and compound-protein-centric databases [22]. Protein-centric databases primarily offer functional and structural information about proteins. The most widely utilized protein-centric databases are the Universal Protein Resource Knowledgebase (UniProtKB) and the Protein Data Bank (PDB). UniProtKB is divided into UniProtKB/Swiss-Prot and UniProtKB/TrEMBL [23]. UniProtKB/Swiss-Prot maintains manually annotated and experimentally verified protein sequences, while UniProtKB/TrEMBL contains computationally analyzed sequences awaiting manual annotation.

As of November 2023, UniProtKB/Swiss-Prot comprises 570 420 meticulously curated sequence entries sourced from 295 467 unique references with a total of 206 321 560 amino acids (UniProtKB/Swiss-Prot release 2023_05). Meanwhile, UniProtKB/TrEMBL featured 251 131 639 sequence entries, comprising 88 223 298 202 amino acids (UniProtKB/TrEMBL release 2023_05). In comparison, the PDB currently contains 211,377 entries corresponding to experimentally determined three-dimensional structures of biological macromolecules, primarily proteins and nucleic acids [24].

On the other hand, compound-centric databases offer comprehensive data about chemical compounds, including their chemical structures, properties, interactions, and bioactivity. Two notable representative compound-centric databases are DrugBank [25] and ChEMBL [26].

Our focus is on compound-protein-centric databases, which provide information on both proteins and compounds and their interactions. The most commonly used compound-protein-centric databases for BAP in the literature are the PDBbind [27] (including PDBbind_core_2013 [28-31] and PDBbind_core_2016 [32]), DAVIS [28], BindingDB [29], and Kiba [30]. The analysis of these data in the following section aims to assist researchers in making well-informed choices when selecting suitable datasets for training and testing DL models designed for BAP tasks.

### 3.1 PDBbind

**Fig. (3):**
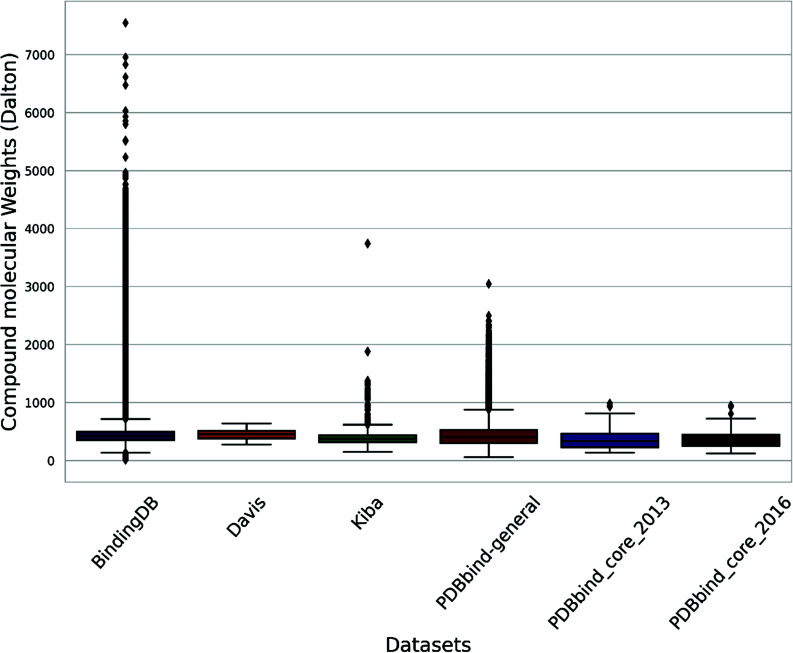
Box plot of compound molecular weights in commonly used datasets.

**Fig. (4):**
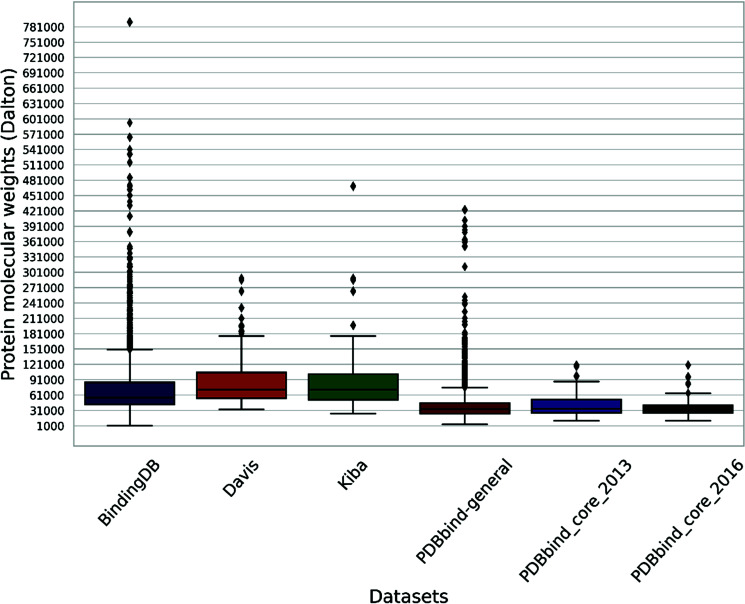
Box plot of proteins molecular weights in commonly used datasets.

The protein molecular weight within the general set spans from 3494 Daltons (Da) to a maximum of 423,549 Da (Figs. **3** and **4**). In contrast, the molecular weights of the compounds range from 57 Da to 3046 Da (Fig. **3**). Outliers for protein molecular weight exceed 75,866 Da, while the upper bound for compound weight is 877 Da.

**Fig. (5):**
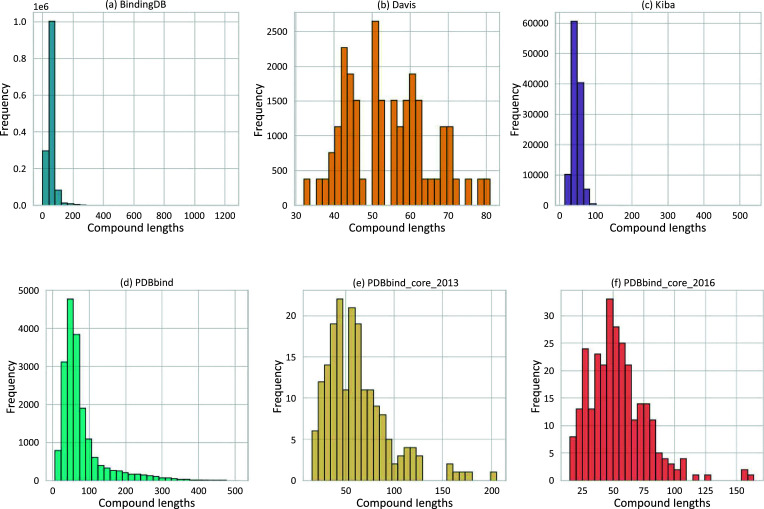
Histograms of compound lengths in commonly used datasets: (**a**) Binding DB; (**b**) Davis; (**c**) Kiba; (**d**) PDBbind General set ; (**e**) PDBbind_core_2013; (**f**) PDBbind_core_2016.

**Fig. (6):**
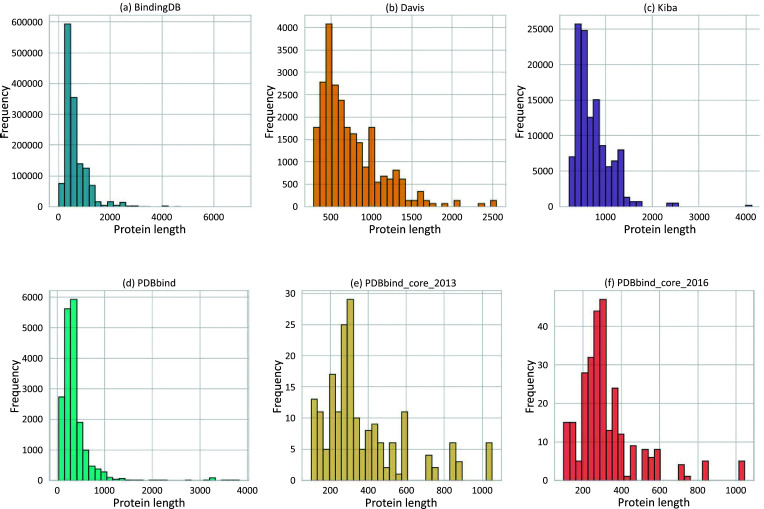
Histograms of protein lengths in commonly used datasets: (**a**) Binding DB; (**b**) Davis; (**c**) Kiba; (**d**) PDBbind General set; (**e**) PDBbind_core_2013; (**f**) PDBbind_core_2016.

**Fig. (7):**
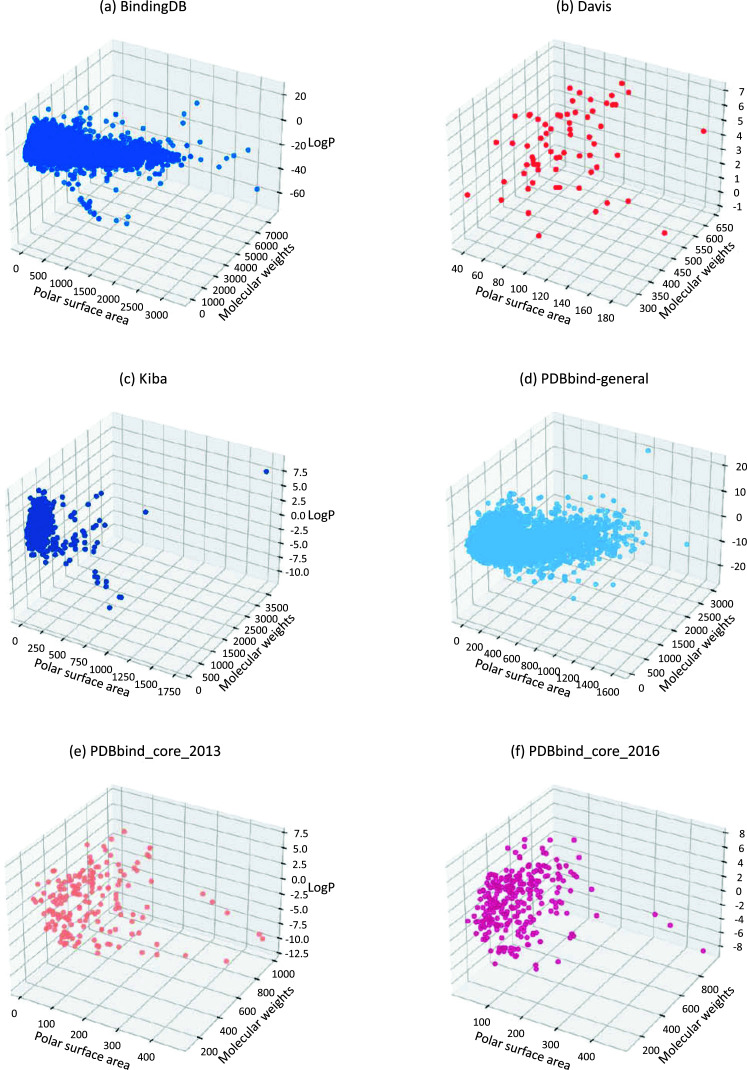
(**a-f**) 3D Scatter plot illustrating the relationship between polar surface area, molecular weight, and LogP of compounds in commonly used datasets.

Most BAP models primarily rely on sequence-level features, and the length distribution of these sequences significantly affects model performance. Identifying the optimal input sequence length during model training is crucial. In the general set, SMILE lengths of compounds vary from 6 to 510 characters (Fig. **5**), while protein sequences range from 31 to 3,833, with outlier thresholds set at 666 for proteins and 147 for compounds (Fig. **6**). When visualizing the compound's polar surface area against molecular weight and the octanol-water partition (LogP), similarities emerge between this dataset and compounds in the BindingDB dataset (Figs. **7a** and **7d**), likely due to overlapping molecules.

#### 3.1.1 PDBbind Core_sets 2016 and 2013

The PDBbind core set is a curated collection of high-quality protein-ligand complexes used to validate and benchmark BAP methods. This essential set is the primary test set for the renowned Comparative Assessment of Scoring Functions (CASF) benchmark. The latest version, referred to here as PDBbind core_2016, is made of 285 proteins‒ligand complexes, much more than the older version PDBbind core_2013, which contained 195 complexes.

A box plot analysis reveals that protein molecular weights in both sets range from 10,000 Da to 121,000 Da (Fig. **4**), while compound molecular weights fall between 121 Da and 950 Da (Fig. **3**). The mean compound molecular weight is 351 Da for core_2016 and 365 Da for core_2013.

The length distribution plots for both sets show diverse ranges of compound SMILE lengths. In core_2016, lengths vary from 15 to 164 (Fig. **5f**), while in core_2013, they range from 15 to 205 (Fig. **5e**). Similarly, protein lengths show variability, ranging from 99 to 1045 for core_2016 (Fig. **6f**) and 99 to 1052 for core_2013 (Fig. **6e**).

#### 3.1.2 PDBbind-koff-2020

A more curated set introduced by Liu *et al.*, known as PDBbind-koff-2020 [33], offers a comprehensive collection of 680 protein-ligand complexes, each characterized by experimental dissociation rate constants (koff). PDBbind-koff-2020 encompasses 155 protein types with dissociation rate constants spanning ten orders of magnitude. The protein molecular weights vary from 11,780 Da to 378,000 Da, with 75% falling below 84,660 Da (Fig. **4**). Additionally, the histogram plot illustrates protein sequence lengths ranging from 99 to 3,390 (Fig. **6g**).

A concluding remark about the PDBbind datasets is the need for careful consideration. Some protein structures provided may contain issues, such as missing coordinates for certain residues or the presence of unusual residues. Moreover, there is significant overlap among the general, refined, and core sets. Due to the high occurrence of low-quality protein-ligand complexes in the general set, it is deemed more suitable to train a DL model using the refined set [34]. Within this refined set, complex structures and binding affinity values have been meticulously curated, thereby ensuring a higher data quality.

## 3.2 Davis

The Davis dataset, introduced by Davis *et al.* [28], is a collection of small-molecule kinase inhibitors and their interactions across the human protein kinome. It includes 72 known kinase inhibitors tested against a panel of 442 kinase assays, covering more than 80% of human protein kinase domains. It provides information on approximately 30,056 drug-target pairs measured in Kd.

The set contains proteins with molecular weights between 32,990 Da and 290,000 Da, as shown in Fig. (**4**). The compounds have molecular weights varying between 275 Da and 650 Da, as depicted in Fig. (**3**). Protein sequence lengths span between 288 and 2549 (Fig. **6b**), while compound lengths range from 32 to 81 (Fig. **5b**).

A scatter plot of the polar surface area, compound molecular weights, and LogP reveals a close association to the chemical space of the PDBbind core_2013 dataset (Figs. **7b** and **7e**). Both chemical spaces are spread out compared to the clustered space of the Binding DB and PDBbind-general sets.

A notable observation regarding the Davis dataset is the presence of overlapping drug-target pairs with other datasets, namely the Metz [35] and Anastassiadis [36] datasets. The Davis dataset is reported to be sharing 2575 drug-target pairs with the Metz dataset and 4255 with the Anastassiadis dataset [30].

## 3.3 BindingDB

BindingDB is a rich and diverse repository of experimentally determined protein-ligand binding affinities. The extensive collection encompasses approximately 2.8 million entries, covering around 1.2 million unique compounds and 9200 distinct proteins. The binding affinity data are expressed as Kd, Ki, or IC_50_.

A significant subset of this dataset, totaling 1.297 million entries associated with 594,000 compounds and 4500 proteins, has been curated from scientific articles by the BindingDB team. Additionally, BindingDB integrates entries from reputable databases, such as ChEMBL and PubChem, along with information drawn from patents. BindingDB offers a user-friendly web interface, facilitating searches based on protein names, ligand names, binding affinities, and experimental methods.

The molecular weight distribution within a subset of the BindingDB dataset, comprising 1.4 million entries, shows that 75% of compound molecular weights fall below 498 Da (Fig. **3**) and protein molecular weights below 86,000 Da (Fig. **4**). (Figs. **5a** and **6a**) demonstrate that both compound and protein lengths have a positively skewed distribution. Figs. (**7a** and **7d**) indicate a close similarity between the compounds of BindingDB and those of PDBbind in terms of their polar surface area, molecular weights, and LogP.

## 3.4 Kiba Dataset

KIBA (Kinase Inhibitor Bioactivity) is another commonly used dataset in BAP. It is a collection of bioactivity data related to kinase inhibitors, offering information about their interactions with various kinase targets. KIBA data integrate diverse sources, including large-scale biochemical assays, to provide a more holistic and accurate representation of kinase inhibitors bioactivity.

Containing data from three major assays and complemented by a novel model-based integration approach, this dataset contributes significantly to understanding the selectivity profiles of kinase inhibitors. It enhances the reliability of drug-target interaction classification by effectively addressing data heterogeneity and leveraging a combination of IC_50_, Ki, and Kd measurements into a single score, known as the KIBA score, which represents the binding affinity.

KIBAs compounds have molecular weights between 150 and 3740 Da (Fig. **3**), while protein molecular weights range between 24,942 and 469,084 Da (Fig. **4**). Length distribution analysis indicates that most compounds have lengths between 14 and 100 (Fig. **5c**), while over 75% of protein lengths are below 915 (Fig. **6c**). Fig. (**7**) shows differences in KIBAs chemical space compared to PDBbind, BindingDB, and Davis.

## 4 COMMON DATA PREPROCESSING METHODS

### 4.1 Data Integration

BAP often requires integrating data from various databases to create a comprehensive and diverse dataset. The process involves several steps to ensure the quality, consistency, and reliability of the integrated dataset. The initial step in building a BAP model consists in gathering information on protein-ligand interactions from various datasets, such as those discussed in Section 3. It is essential to carefully select data encompassing a diverse range of protein-ligand interactions (PLIs) to capture different structural and chemical characteristics. This diversity is necessary for building robust and generalizable predictive models.

When performing data integration, it is crucial to establish common identifiers for matching entries across various databases. This involves aligning entries based on shared identifiers, such as protein or compound names, as well as specific identifiers, such as PDB identifiers for proteins in the PDB database, UniProt accession numbers for the UniProt database, or InChIKeys for compounds. Due to varying data acquisition and curation practices, different databases may employ inconsistent naming conventions or data representations.

### 4.2 Affinity Measures Conversion (IC_50_, KD, KI)

Converting affinity measures is crucial in building an accurate and generalizable DL model for BAP. The necessity to transform the affinity values arises from the diverse ways in which experimental assays express binding affinity, typically in terms of Kd, IC_50_, or Ki. These represent different aspects of the binding strength between a protein and a ligand. Using different units across datasets introduces challenges in comparing and integrating data for model training and evaluation. For instance, data from BindingDB comprise a multitude of assays, each adopting its preferred metric for binding affinity.

The variability in unit measurements can result in inconsistencies and inaccuracies within model predictions. Researchers often resort to strategies for converting affinity values to address the issue. One approach, as exemplified by DeepLPI [37], involves selecting a specific unit, such as Kd, and retaining only interactions represented in that unit. The approach ensures a consistent representation of binding affinity throughout the dataset.

In the case of MAPL-FMP [38] working with the PDBbind dataset, they considered instances with activity expressed in terms of either Ki or Kd while excluding those expressed in IC_50_ or EC50 values.

In their work on the PDBbind dataset, Shen *et al.* (2021) used the binding affinity expressed in the log space of the original units (−Log10Kx). Similarly, other models, such as DeepDTA [39], DeepGLSTM [40], and DeepCDA [41], employ a similar approach for datasets such as the Davis dataset. The affinity values were converted into PKD values using Eq. (1), where Kd is the dissociation constant. This conversion ensures that diverse datasets with varying affinity units can be effectively integrated into the model training process.


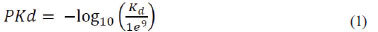


### 4.3 Fixing optimal Sequence Length for Proteins and Ligands

In BAP, the length of both protein and ligand sequences can vary significantly, as described in Section 3. The variability poses a challenge for DL models, which typically require fixed-size inputs. A maximum length constraint for protein and ligand sequences is often implemented to address the issue. By implementing maximum length constraints, computational efficiency, generalizability, and performance can be improved. The specific length selection is guided by the datasets characteristics, the complexity of the binding interaction, and the available computational resources.

Determining the optimal length for protein and ligand sequences involves a balance between capturing sufficient information and maintaining computational efficiency. Table **1** lists the maximum length constraints used in various studies. The common choice for protein sequence length is 1000-1200, while the ligand sequence length typically ranges from 85 to 200.

### 4.4 Proteins and Ligands Representation

#### 4.4.1 Ligands Representation

Diverse data formats and encoding schemes are employed to represent ligand compounds and proteins. Ligand compounds are commonly represented using Simplified Molecular Input Line Entry System (SMILES) notation, fingerprints, and molecular graphs.

SMILES are textual representations of chemical structures providing a concise and human-readable format for representing molecular structures. They encode information about atom types, bond types, and connectivity in a linear string. Widely utilized in literature, SMILES serves as the predominant representation of ligands. This textual representation is subsequently encoded using one-hot or label encoding techniques to facilitate its integration into DL models. The advantages of SMILES include their simplicity, ease of interpretation, and compatibility with various cheminformatics tools. However, SMILES have limitations, such as not capturing 3D spatial information and being sensitive to variations in molecular representations [42-53].

Fingerprint representations are also common. Many studies have successfully used molecular fingerprints to represent ligands. Fingerprints are binary vectors representing the presence or absence of specific substructures within a molecule. They efficiently capture key structural features and are suitable for similarity searching and ML tasks. For instance, RFDT [54] utilizes the PubChem substructure fingerprint made of 881 bits [55] to represent ligand compounds. Wang *et al.* (2021) investigated a variety of protein-ligand interaction fingerprints (IFPs) for BAP. They found that atom pair count-based and substructure-based IFPs performed well in target-specific and generic scoring tasks, demonstrating their potential for improving BAP accuracy [55]. However, fingerprint representations have limitations, such as the possibility of not capturing all relevant substructures and the variation in encoding schemes between different algorithms.

In addition to SMILES and fingerprints, the molecular graph is another common format for representing ligands. They represent molecules as graphs, where nodes represent atoms and edges represent bonds. Molecular graphs offer an advantageous format for ligand representation. They capture both structural and topological information, allowing the incorporation of 3D spatial information. This format has been employed in various proposed models, including DeepGLSTM, GraphscoreDTA [56], DGraphDTA [57], and GraphDTA. While suitable for graph-based ML approaches, molecular graphs can be computationally expensive for processing large molecules.

In addition to the three major formats mentioned above, various other data formats are available for representing ligand compounds, each with advantages and disadvantages. For instance, Pafnucy [58] utilized 3D coordinates, explicitly capturing atomic positions in space. While this provides detailed structural information, it demands substantial computational resources.

#### 4.4.2 Proteins Representation

Like ligand representation, various data formats and encoding schemes are used for proteins, including primary amino acid sequences, secondary structures, and graphs. Proteins primary amino acid sequence is a fundamental representation, encoding the linear arrangement of amino acids forming the protein chain. This representation is widely employed due to its simplicity and direct connection to the genetic code. In studies such as ML-DTI, DeepDTAF, Multi PLI, and CAPLA, protein sequences are encoded using methods such as one-hot encoding, where each amino acid is represented as a binary vector. WideDTA, ColdDTA, and PCNN-DTA [50] employed label encoding, assigning each unique amino acid a specific numerical label. While primary sequences offer insights into the linear structure of proteins, they may not capture the spatial relationships crucial for binding interactions.

Secondary structure representations also provide information about local folding patterns in proteins, including alpha-helices, beta-sheets, and loops. These representations are often derived from experimental methods like X-ray crystallography or predicted using algorithms such as DSSP (Dictionary of Secondary Structure of Proteins)) [59]. In MAPL-FMP [38] and CAPLA, protein secondary structures are calculated using the DSSP algorithm and incorporated along with the physicochemical properties of residues for comprehensive protein representation.

For a more detailed and spatially aware representation, proteins can be represented as graphs. In GraphscoreDTA and Lim *et al.* [60], the 3D structures of proteins were used to construct protein graphs, where nodes represent atoms and edges represent interactions. This representation preserves spatial relationships, contributing valuable information for binding affinity prediction. However, working with 3D structures can be computationally demanding, especially for large proteins.

In addition to these representations, contact maps derived from sequence alignment and predicted using methods such as Pconsc4 are utilized in ColdDTA, DGraph-DTA, and GSAML-DTA [61]. These maps capture residue-level interactions, offering a different perspective on protein structure and function.

In summary, the choice of data format for compounds or proteins depends on several factors, such as the desired level of detail and the computational resources available. Understanding the various options and their trade-offs allows one to choose the optimal representation for specific needs.

## 5 DEEP LEARNING MODELS FOR BAP

Selecting a suitable model becomes crucial after data preprocessing steps and appropriate data representation. The chosen model should effectively learn feature representations and extract valuable information to highlight the interactions between compounds and proteins, consequently enabling accurate binding affinity prediction. DL has emerged as a powerful tool in this domain, demonstrating the ability to learn complex patterns from extensive datasets and provide accurate predictions. Recent publications and trends suggest that DL models for BAP can be broadly classified based on their chosen feature extraction and learning model. Notably, CNNs, RNNs, GNNs, and transformer-based models emerge as the most prevalent choices. This section delves into a detailed exploration of the application of these models to BAP, with a focus on their respective strengths and limitations.

### 5.1 CNN-based Models

CNNs are inspired by the human visual system [62] and have proven highly effective in image processing [63-66]. They operate through convolutional, pooling, and fully connected layers, learning hierarchical features ranging from simple edges to complex patterns. Initially prominent in image classification and object detection, CNNs have expanded into chemogenomic methods by adapting to different data types. To accommodate various data formats, 1D CNNs, 2D CNNs, and 3D CNNs have evolved with specialized architectures tailored to the nature of the input data.

#### 5.1.1 1D CNN

1D CNN, or one-dimensional convolution neural network, is a fundamental building block in DL architectures designed for sequential data processing. Tailored for one-dimensional sequences such as time series or text, 1D CNN operates by sliding a filter or kernel along the input sequence, capturing local patterns and relationships within the data. This ability to capture local patterns and dependencies in sequential data makes it valuable in various applications, including speech recognition, sentiment analysis, and computational biology.

1D CNN has been extensively used for BAP because of its suitability for sequential data such as compound SMILES or protein amino acid sequences. In their work, Öztürk *et al.* [39] introduced DeepDTA, the first model to utilize 1D CNN for leveraging raw sequence information, employing SMILES for compounds and primary sequences for proteins. With its simple architecture in the Y shape using simple compound and protein representations, DeepDTA demonstrated improved predictive capabilities compared to established baselines such as KronRLS [67] and SimBoost [68]. Although DeepDTA’s studies represent a significant advancement in utilizing CNNs for molecular sequence representation, they also acknowledge limitations, suggesting exploring alternative approaches, such as long short-term memory (LSTM), for more effective protein sequence representation. Using the same model as DeepDTA, AttentionDTA [69] introduces a novel approach to drug-target interaction prediction by incorporating an attention mechanism to enhance binding affinity predictions. While maintaining the use of the same protein and ligand representations as input, AttentionDTA leverages the power of attention mechanisms to focus on critical subsequences within proteins and drugs selectively. The attention-based enhancements, coupled with the use of 1D-CNN, lead to superior performance compared to DeepDTA.

DeepDTAF [70] presented a comprehensive approach to BAP by integrating diverse input representations and employing a 1D-CNN-based architecture. They integrated protein sequences, pocket sequences, and protein structural properties. For input representation, the method employed label encoding for ligand SMILES and OneHot encoding for amino acid sequences, resulting in rich and informative feature vectors. The proposed model architecture incorporated dilated convolution for proteins and ligands while using traditional convolution for pocket sequences. The model emphasized the importance of both local and global features and highlighted the significance of capturing interactions within binding pocket sequences and entire protein sequences.

Expanding upon the character-based DeepDTA approach, WideDTA [46] introduces a word-based model for representing protein and ligand sequences in BAP. Due to the non-sequential nature of motifs and domains in protein sequences, as well as the potential for overlapping residues in both motifs and maximum common substructures (MCS), they argue for the use of a word-based model over a character-based model. Employing a 1D CNN architecture, the authors integrated ligand SMILES, ligand maximum common substructure (LMCS), protein sequences, and protein motifs and domains as input features into the WideDTA model. Majumdar *et al.* [71] presented a 1D-CNN-based framework for ligand prediction against the S-glycoprotein of SARS-CoV-2. The input representation involves the fusion of protein sequence composition descriptors (PSC) (Lee *et al.*, 2019) and ligand-extended connectivity fingerprints (ECFP4). PSC descriptors capture essential features of protein sequences, comprising amino acid composition (AAC), dipeptide composition (DC), and tripeptide composition (TC). Zhu *et al.* (2023) introduced FingerDTA (X. Zhu *et al.*, 2023), a fingerprint-embedding framework for BAP. Using 1D-CNN and fully connected layers, FingerDTA integrates one-hot encoded protein and ligand sequences with global information represented by fingerprints generated from the whole sequence of drugs and ligands. In PCNN-DTA [50], the authors introduce the pyramid network convolution drug-target binding affinity, utilizing multiple 1D-CNN layers through a feature pyramid network (FPN). PCNN-DTA incorporates a 1D-CNN-based architecture within the FPN, allowing the extraction of hierarchical features from drugs and proteins represented as SMILES and primary sequences, respectively. The bottom-up pathway of the FPN employs residual 1D-CNNs to capture detailed information, while the top-down pathway enhances representation with deconvolution layers. The use of 1D-CNNs at various levels of the FPN enables the retention of both low-level and high-level information, contributing to improved binding affinity prediction accuracy.

While using readily available 1D representations such as SMILES for ligands and primary sequences for proteins offers certain advantages in computational efficiency and data handling, its application in predicting protein-ligand binding affinity comes with inherent limitations due to the complex nature of the interaction. The reliance on string-based representations may lead to the loss of crucial structural information, compromising the models predictive accuracy and diminishing the functional relevance of the learned latent space. Additionally, proteins and ligands are not rigid entities; they exhibit conformational flexibility, adopting different shapes that influence binding [72]. 1D models lack the ability to account for this dynamic behavior, potentially overlooking key conformations crucial for the interaction. These limitations underscore the importance of exploring alternative representations that better capture structural information, thereby enhancing the overall performance and interpretability of BAP models.

#### 5.1.2 2D CNN

A two-dimensional convolutional neural network, commonly known as 2D CNN, is a DL architecture designed for processing and analyzing structured grid data, such as images. Unlike its one-dimensional counterpart, the 2D CNN operates on two-dimensional input data, allowing it to capture spatial hierarchies and patterns within the input. This type of neural network has proven highly effective in image-related tasks due to its ability to recognize spatial relationships, edges, and hidden features. In BAP and molecular analysis, 2D CNNs represent a significant advancement beyond the constraints of 1D representations. They excel in handling input features such as intermolecular descriptors, structural information, and physicochemical properties, all of which can be effectively represented in two dimensions. This encompasses descriptors such as contact maps and evolutionary information.

OnionNet [73] proposed a 2D CNN for BAP. The authors input representation relied on rotation-free element- pair-specific contacts grouped into distance ranges. This resulted in a dataset with 3840 features transformed into a two-dimensional tensor to leverage the power of 2D CNNs. Similarly, Wang *et al.* (2021) presented OnionNet-2, which used 2D CNN to extract features from residue-atom contacting shells. The number of contacts in multiple distance shells characterized the interactions between protein residues and ligand atoms. Shim *et al.* (2021) introduced SimCNN-DTA [74]. The model applied a 2D CNN to the outer product of column vectors derived from Tanimoto and Smith-Waterman similarity matrices for drugs and targets, respectively.

The workflow involved calculating similarity matrices, computing outer products, and utilizing a 2D CNN to extract deep features and predict binding affinities. In MDeePred [75], a multichannel protein featurization approach for BAP was proposed. The authors integrated diverse protein features, including sequence, structural, evolutionary, and physicochemical properties, into multiple 2D vectors to create comprehensive protein representations. MDeePred adopts a proteochemometric approach, utilizing compound and target protein features at the input level to model their interaction. The proposed method leverages a 2D-CNN architecture for the target protein side, where protein feature matrices are fed as input channels. The compound side employs a feed- forward neural network with circular molecular fingerprints as input. The outputs of the protein-side CNN and the compound-side neural network are combined in a pairwise-input hybrid deep neural network, leading to the binding affinity prediction.

The authors of MPS2IT-DTI [76] encoded molecule and protein sequences into images for BAP. They converted the molecules SMILES and amino acid sequences into matrices of numerical values, creating unique visual signatures for each sequence. The mapping process involved defining k-mers [77, 78], creating and normalizing counting vectors, and reshaping them into image matrices. Subsequently, the deep neural network of MPS2IT-DTI employed a dual 2D-CNN for both molecule and protein inputs to predict the binding affinity.

Although 2D CNNs have demonstrated promising outcomes in various proposed models, they also have certain drawbacks. One significant drawback arises from their inherent design, which is focused on 2D grids, potentially hindering their ability to fully capture the spatial complexity of three-dimensional intermolecular structures between proteins and ligands. The projection onto 2D grids may result in the loss of crucial spatial information critical for understanding molecular interactions. Furthermore, in contrast to 1D CNNs, 2D CNNs are not well suited for modeling sequential dependencies in proteins and ligands, which are essentially composed of amino acids and atoms [79]. These networks might inadequately capture long-range dependencies that may be essential for accurate predictions. Additionally, the performance of 2D CNNs is highly sensitive to the chosen input representations, such as molecular descriptors or features [80]. Inaccurate or insufficiently informative input representations can impede the models ability to discern relevant patterns, affecting its predictive accuracy.

#### 5.1.3 3D CNN

In contrast to 1D and 2D CNNs, 3D CNNs can leverage the three-dimensional structural information of molecular complexes. Here, intermolecular structures are transformed into 3D grids or voxel representations. Each voxel corresponds to a specific region in three-dimensional space. These grids effectively contain crucial information regarding the spatial arrangement of atoms, their properties, and their interactions.

Employing a 3D grid representation for molecular complexes, Pafnucy [58] utilized a 3D CNN architecture to extract spatial features. The input, structured as a 4D tensor, incorporated cartesian coordinates and atom feature vectors. Pafnucy surpassed classical scoring functions, including the X-Score [81], when evaluated on benchmark datasets like the PDBbind core set 2013 and Astex [82]. Pafnucy underscores the effectiveness of 3D CNNs in directly learning relevant features from the structural information of the complexes.

KDEEP [83] adopted a 3D CNN architecture inspired by SqueezeNet, tailored for 3D convolution tasks. The networks design simplified depth due to constraints related to training sample size and image resolution while maintaining other architectural aspects, including the use of rectified linear units (ReLUs) as activation functions. In KDEEPs input representation, descriptors adapted from prior work are used to represent proteins and ligands. The representation involves a 3D voxel scheme based on the van der Waals radius for each atom type, encompassing properties such as hydrophobicity, hydrogen bonding, aromaticity, and ionizability.

In Sfcnn [84], the authors adopted a concise featurization method, representing the protein-ligand complex with a 3D grid or 4D tensor. Unlike Pafnucy and KDeep, Sfcnn’s approach simplifies atom featurization, extracting only basic atomic type information. The 3D CNN architecture was designed to handle the transformed input, and the model was evaluated on various datasets, outperforming other scoring functions. The authors highlighted the interpretability of the model through Grad-CAM, making intermediate layers of the neural network more understandable.

DeepAtom [85] utilized a 3D-CNN architecture to extract binding-related atomic interaction patterns from voxelized complex structures. The input representation in DeepAtom consisted of rasterizing protein-ligand complexes into a 3D grid box centered on the ligand. Each voxel in the grid box contains several input channels representing different raw information of atoms located around the voxel. The authors employed a lightweight 3D-CNN model to hierarchically extract useful atom interaction features supervised by the binding affinity score. The model architecture comprised three building blocks: the atom information integration block, the stacked feature extraction block, and the global affinity regression block. The atom information integration block utilizes a pointwise convolution layer and a 3D max pooling layer to fuse atom information across different channels and increase translational invariance.

Inspired by the ResNext architecture, AK-Score [86] employed an ensemble of 3D-CNN models, demonstrating improved prediction accuracy compared to previous models. The network architecture comprised 15 stacked layers of an ensemble-based residual layer (RL) block. Each RL block consisted of three stacks of convolutional layers: batch normalization, rectified linear unit (ReLU) activation, and residual summation, facilitating parallel processing of the input tensors.

Limited generalization due to the scarcity of high-quality and unbiased labelled data poses a significant challenge for 3D CNNs in BAP [87, 88]. The available datasets may not adequately represent the diverse landscape of PLIs. Consequently, models trained on such data may struggle to generalize well on unseen protein-ligand pairs, hindering their predictive accuracy and real-world applicability [89].

Moreover, the memory usage and the computational cost required for 3D CNNs add another layer of complexity to the BAP. The computational demands of these voxel-based approaches escalate rapidly with spatial resolution, leading to significant memory usage and computational costs [90]. As a result, the computational overhead associated with 3D CNNs may limit their scalability and practical utility, particularly in high-throughput screening scenarios where efficiency is paramount. Table **2** presents the summary of CNN-based models for BAP.

### 5.2 RNN-based Models

A Recurrent Neural Network (RNN) [31] is a neural network architecture designed to process sequential data by maintaining hidden states that capture dependencies between elements in the sequence. Unlike traditional feedforward neural networks, RNNs have feedback loops that allow information to persist over time, making them well-suited for tasks where the order of elements matters, such as natural language processing, time series analysis, and speech recognition. This architecture offers several advantages over CNNs, including temporal dependency modeling, adaptability to variable-length sequences, adaptive feature extraction from sequential data, and computational efficiency. RNNs and their variants have been extensively used in computational biology for tasks ranging from sequence analysis [32] to structure prediction [33-35].

#### 5.2.1 LSTM-based Models

To address the vanishing gradient problem that occurs when training standard RNNs on long sequences, specialized variants such as long short-term memory (LSTM) networks were introduced. As shown in Eq. (2), the LSTM unit updates its cell state. *c_t_* and hidden state *h_t_* based on the current input *x_t_*, the previous hidden state *h_t-1_*, the previous cell state *c_t-1_*, and the gating mechanisms. This mechanism allows the LSTM to retain information over long sequences and excel in tasks reliant on long-term dependencies.


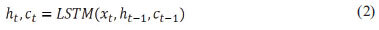


In DeepCDA [6], LSTM is utilized as part of the model architecture to predict the binding affinity of compound-protein pairs. The LSTM network is combined with CNN layers to learn representations of both compounds and proteins. The raw protein sequences and compound SMILES strings are initially fed into the model as inputs. Subsequently, CNN is used to extract mid-level features hierarchically before passing them to the LSTM, which encodes the sequences dependencies. Additionally, the model incorporates a two-sided attention mechanism to capture the interaction strength between each protein substructure and compound substructure pair. Meanwhile, the authors of DGDTA [36] modified the GraphDTA model for BAP by introducing a triple-channel model in which LSTM was used to encode the proteins features.

DeepGLSTM [9] integrated a graph convolutional network (GCN) and bidirectional long short-term memory (Bi-LSTM) layers to predict the binding affinity between FDA-approved drugs and viral proteins of SARS-CoV-2. The GCN layers processed drug compounds while the Bi-LSTM layer processed the protein sequences, leveraging its bidirectional nature to capture temporal dependencies effectively.

#### 5.2.2 GRU-based Models

Another variant is the Gated Recurrent Unit (GRU), which simplifies the LSTM architecture by combining the forget and input gates into a single update gate. GRU has been shown to be effective for BAP. To overcome the drawbacks associated with the loss of crucial compound information observed in GCN-based models, Wang *et al.* introduced SSGraphCPI [37], a three-channel DL framework. SSGraphCPI integrates a seq2seq model based on a GRU with an attention mechanism and graph convolutional neural networks (GCNNs). By leveraging GRU-based seq2seq architecture, the model translates input sequences into fixed-dimension vectors termed Thought Vectors, thereby enabling the effective representation of compound and protein features. In GDGRU-DTA [38], the authors proposed to enhance the GraphDTA model by incorporating the gated recurrent unit (GRU) and bidirectional gated recurrent unit (BiGRU) for interpreting protein sequences. The integration aimed to capture long-term dependencies in protein sequences more effectively. ResBiGAAT incorporated both physicochemical properties and sequence-level features of proteins and ligands to enhance prediction accuracy. The model employed a multi-layered Residual Bi-GRU coupled with two-sided self-attention mechanisms to capture long-term dependencies within sequences, achieving competitive performance in predicting the binding affinity.

The inherent drawback of the RNN-based models lies in their limited feature representation of proteins and compounds. As a result, they may fail to capture all aspects of PLIs that influence binding affinity. The lack of comprehensive representation can hinder their ability to predict binding affinity in real-world scenarios accurately. Furthermore, these models often suffer from a lack of generalizability, making them less practical for applications in predicting binding affinity.

### 5.3 Transformer-based models

Transformers, including variants like BERT and GPT, are renowned for their effectiveness in sequence-to-sequence tasks, such as machine translation and text generation [39]. Transformers recently gained traction in biomedical and pharmaceutical research, including drug discovery. They have been successfully applied in various tasks such as molecular property prediction [40-42], protein structure prediction, sequence analysis [43-45], and genome analysis [46-48].

Hu *et al.* [49] combined a transformer and a graph attention network (GAT) to reduce data sparseness and computational costs while achieving better accuracy. The Transformer encoder processed the protein sequence, while the GAT handled the protein contact map and drug embeddings. This approach enabled the model to effectively capture the structural information of proteins and drugs, leading to improved performance compared to traditional methods. TranDTA [50] investigated BAP through diverse protein input feature techniques, including UniRep [51], ProtBert, and ProtAlbert [52]. Compound drugs were represented using molecular fingerprints and RoBERTa-encoded SMILES sequences [53]. The authors found that using ProtAlbert for protein representation and molecular fingerprint representation for drugs produced optimal results. DTITR [54] employed two parallel Transformer-Encoders for proteins and compounds, incorporating self-attention and cross-attention layers to predict binding affinity effectively. In PLAPT [55], the authors leveraged transfer learning from pretrained transformers such as ProtBERT [52, 56], and ChemBERTa [57], utilizing protein primary sequences and SMILES notation for ligands. The approach enabled them to achieve high accuracy while minimizing computational resources, highlighting the importance of utilizing pretrained models in computational drug discovery.

While transformers offer significant advantages over CNN models in capturing complex patterns, there are some constraints inherent to their architecture and functionality. Transformer-based models typically require large amounts of labeled data for effective training. However, obtaining labeled data for compound-protein interactions can be challenging and may limit the models ability to generalize well to unseen interactions or compounds. Furthermore, transformers may struggle to generalize effectively across diverse compound-protein interaction datasets due to variations in compound structures, protein conformations, and binding affinities. Differences across datasets can pose significant challenges for transformers in learning robust representations that generalize across different domains. Additionally, transformers, especially large-scale models like ProtBert and ProtAlbert, are computationally intensive and require substantial resources for training and inference. The complexity can pose challenges in terms of computational cost and scalability, particularly for large datasets or real-time applications.

### 5.4 Graph-based Models

Graph neural networks (GNNs) [58] have emerged as powerful tools in drug discovery and computational biology, particularly in binding affinity prediction. Traditional methods often rely on sequence-based or structure-based approaches, which may overlook molecular interactions and fail to capture the complex relationships between molecules. GNNs offer a unique advantage by enabling the representation of molecular structures as graphs, where atoms or residues serve as nodes and chemical bonds or interactions as edges. The graph-based representation allows GNNs to capture the spatial and functional relationships between atoms or residues more effectively, thus enhancing the accuracy of binding affinity prediction.

In terms of input representation, some authors represent only drug molecules as a graph while maintaining the primary sequences of the target proteins. Examples include GraphDTA [4], DeepGLSTM [9], GDGRU-DTA [38], DeepGS [59], and EmbedDTI [60]. In these approaches, the drug molecules are represented as graphs to capture their structural features, while the primary sequences of the proteins are processed separately to extract relevant information. Conversely, some authors represent drug molecules and the target proteins as graphs. Examples include Dgraph-DTA [61], X-DPI [62], and WGNN-DTA [63]. These methods construct graphs for both the drug molecule and the protein, allowing for the integration of structural and interaction information from both entities.

Various GNNs have been proposed to address the complexities inherent in BAP. Among these models is the Graph Attention Networks (GAT), which assign varying weights to neighboring nodes, enabling focused attention on important features during message passing. DGDTA [36] introduced a dynamic graph attention network combined with Bi-LSTM to enhance binding affinity prediction (BAP). The model integrated dynamic graph attention by representing drug compounds as graphs and proteins as 1D sequences to capture important features from drug graphs. Additionally, Bi-LSTM processed protein sequences to extract contextual information. By integrating 3D structural information of proteins with 2D graph representations of ligands, PSG-BAR [64] employed a residual graph attention network to predict binding affinity effectively. Their approach enhances the models predictive capabilities by leveraging both the protein structures and molecular graph features of ligands, offering a comprehensive understanding of the complex PLIs.

Another prominent graph architecture used in BAP is the graph convolutional network (GCN) [65], which applies convolutional operations on graph-structured data to aggregate information from neighboring nodes. GCNs excel at capturing local structural features within molecular graphs, providing insights into the complex relationships between atoms or residues. To reduce the computational cost associated with utilizing 3D voxelized grid cubes, Son *et al.* proposed a graph convolutional neural network termed GraphBAR [66]. Their model employs a graph convolutional network converting binding complexes into graphs with the binding site determined by the distance between ligands and proteins. APMNet [67] introduced a cascade graph convolutional neural network model for binding affinity prediction. The architecture incorporates graph convolutional layers, including ARMA [68] and MPNN, to capture molecular graph features effectively and improve predictive performance. In GCAT [69], the attention-enhanced graph cross-convolution network was employed as the model architecture for exploring the binding affinity between drugs and proteins based on the atomic arrangement in three-dimensional space. GCAT consisted of a cross-convolution, which simulated interactions between the protein and the drug through an aggregate-update mechanism and self-attention pooling, utilized for generating graph-level representations.

In addition, graph isomorphism networks (GIN) [70] have also proven to be efficient in BAP by learning graph representations invariant to node ordering. GINs employ message-passing schemes to iteratively update node embeddings, enabling robust modeling of complex molecular structures. Their ability to capture variations in molecular configurations makes them well-suited for predicting binding affinities and elucidating the underlying mechanisms of molecular interactions. GIN was proven effective in the GraphDTA [4] study, demonstrating the best performance across the Kiba and Davis datasets compared to GCN and GAT. Table **3** presents a comprehensive list of various GNNs introduced to predict binding affinity between proteins and ligands efficiently.

### 5.5 Emerging Deep Learning Methods for BAP

In addition to GNNs and transformers, several other emerging DL techniques are being explored for BAP. Some of these include Transfer learning and Reinforcement learning.

Transfer learning techniques leverage pre-trained models or knowledge from related tasks to improve CPI prediction performance, particularly in limited labelled data contexts. By transferring knowledge from tasks with abundant data to tasks with sparse data, transfer learning enhances model generalizability and prediction accuracy. [92-140] explored deep transfer learning to predict drug-target interactions for understudied proteins, showing superior performance for datasets with fewer than 100 compounds compared to training from scratch. In [141], A3C, a novel reinforcement learning technique, notably improved protein-ligand docking predictions for single and multi-atom ligands compared to a basic model. Extension of this approach to binding affinity prediction could yield improved performance.

Another emerging method is the capsule networks, which offer an alternative approach to traditional CNNs by representing hierarchical structures within data. Capsule networks have shown promise in capturing spatial relationships and hierarchical features in molecular structures, making them suitable for CPI prediction [142]. introduced CapBM-DTI, combining capsule networks with pre-trained BERT for target protein sequence extraction and MPNN for compound graph feature extraction, showing good performance and applicability in virtual screening, including for COVID-19 treatment. Despite not directly treating binding affinity as a regression task, these emerging methods show potential for application in binding affinity prediction.

Furthermore, Meta-learning and Few-shot learning methods are gaining attention in the context of CPI. The authors of MetaDTA proposed Meta-learning, a technique focused on developing models that can quickly adapt to new tasks or domains based on previous experiences. MetaDTAs approach leverages Attentive Neural Processes (ANPs) to model binding affinities for each target protein as a regression function of compounds, demonstrating superior performance even with limited data availability [143-151].

On the other hand, ZeroBind authors proposed Few-shot learning, where models are trained to generalize from a small number of labeled examples [152]. ZeroBinds protein-specific zero-shot predictor utilizes subgraph matching for drug-target interactions, achieving remarkable performance, especially for unseen proteins and drugs, and showcasing adaptability even with limited prior information. Integrating these approaches into the landscape of BAP methods could lead to further improvements in predictive accuracy and generalizability, particularly for underrepresented proteins.

## 6 CHALLENGES AND FUTURE DIRECTIONS FOR BAP

Despite significant advancements, several challenges persist, including data quality and availability, model complexity, interpretability and explainability.

### 6.1 Data Quality

While the quantity of available datasets has increased, ensuring their quality remains a persistent challenge. Achieving accurate predictions in BAP heavily relies on the quality and quantity of available datasets [143]. The challenge lies in obtaining comprehensive datasets encompassing a diverse range of molecular interactions. The need for more high-quality data, particularly for specific protein-ligand pairs or underrepresented classes, hampers the ability to train models that generalize well across different independent test sets.

### 6.2 Model Complexity

Deep learning models, while powerful in their predictive capabilities, often exhibit high levels of complexity. For instance, when using a 3D voxel grid representation of protein-ligand complexes to train a 3D CNN, the number of trainable parameters scales with factors like grid size, number of channels per voxel, and the complexity of the convolutional layers. The dimensions of the voxel grid determine the input size, with larger and higher-resolution grids resulting in more input features and, subsequently, more trainable parameters.

Similarly, in GNN, the size of the molecular graph, determined by the number of atoms and interactions, influences the model complexity. The chosen GNN architecture, including the number of layers, message-passing functions, and hidden feature dimensions, also contributes to the total trainable parameters. Simpler GNN architectures with fewer layers and lower dimensions can enhance computational efficiency. Notably, research suggests that effective GNN models can achieve good performance with a relatively small number of parameters [144], making them potentially more scalable for practical applications compared to 3D CNNs with larger parameter sizes associated with voxel grids.

### 6.3 Interpretability and Explainability

Many DL models used for BAP are complex and act as black boxes. Understanding how they arrive at a specific prediction can make the model trustworthy and help to identify potential biases. PLIs involve complex 3D structures and numerous physicochemical properties. Capturing these complexities often leads to high-dimensional data representations that are challenging to interpret directly. Attention mechanisms can highlight specific regions of the protein or ligand that the model focuses on for prediction. This can provide clues about the interatomic interactions driving binding affinity. However, it comes at the cost of making the model more computationally expensive and adding more learnable parameters.

Some emerging techniques, such as Grad-CAM (Gradient-weighted Class Activation Mapping) [145], can help visualize the areas of a protein structure most influential for the models prediction [84, 86]. The visual heatmap can offer insights into the spatial patterns the model considers important. Similarly, the LIME [146] and SHAP [147] techniques can be applied to understand how individual features contribute to specific predictions or the overall model behavior. Even if the feature that contributed to the prediction is known, it is still necessary to understand how it contributed. Hence, it is necessary to make the models more explainable. This underscores the ongoing efforts in the scientific community to develop explainable models tailored to the complexity of BAP.

### 6.4 Integration of Computational and Experimental Approaches

Recent research efforts, particularly in the context of COVID-19, underscore the practical impact of computational methods in drug discovery. For example, at the Experimental Drug Development Centre (EDDC), computational molecular modeling was employed to screen a large library of FDA-approved drugs against the SARS-CoV-2 main protease (3CLpro) [148]. This approach led to the identification of 47 promising candidates based on their computed binding affinities, which were then experimentally validated. Several drugs, including boceprevir and ivermectin, demonstrated significant inhibitory effects on 3CLpro, highlighting how computational predictions can effectively guide experimental drug development. This underscores that while computational methods are invaluable in narrowing down potential drugs, they are most effective when combined with experimental approaches to confirm therapeutic efficacy and safety.

### 6.5 Shifts in BAP Modeling

There has been a noticeable shift in the paradigm for BAP, with graph neural networks emerging as the trending architecture. This transition signifies a move from the conventional use of convolutional neural networks toward adopting graph neural networks. The unique ability of GNNs to effectively model complex relationships within molecular structures has positioned them as a preferred choice in BAP, reflecting the continuous evolution and exploration of innovative GNN architectures in computational biology. Future directions could focus on further enhancing the capabilities and applications of GNNs for BAP. One potential avenue for exploration involves refining GNN architectures to be more explainable and interpretable. This would address the challenge of understanding how GNNs arrive at specific predictions, thus increasing trust in the models and facilitating their adoption in practical settings.

In addition to interpretability, it is essential to ensure that GNNs remain computationally efficient in terms of time and resources without compromising model performance. Optimizing GNN architectures and training procedures can help mitigate computational burdens while maintaining predictive accuracy, enabling widespread adoption across various computational biology applications.

Future directions should focus on refining data splitting strategies and model assessment techniques to address the challenges of imbalanced affinity value distributions and dataset representativeness. By considering protein and compound similarity and affinity value distribution during dataset splitting, researchers can ensure the creation of representative sets of protein-ligand pairs and prevent the overestimation of DL models. This comprehensive approach enhances model robustness, generalizability, and practical applicability in BAP tasks. Additionally, Generative models have the potential to revolutionize BAP prediction by facilitating de novo ligand design and data augmentation. By generating novel molecular structures with optimized binding properties, thus expanding the chemical space available for exploration, these models can improve the generalizability and optimize the BAP algorithm. NeuralPLexer [149] is a valuable example, which predicts 3D structures of protein-ligand complexes, which is particularly beneficial for proteins with limited experimental data. Moreover, the integration of protein structural information beyond the binding pocket into BAP models holds significant potential. Exploring the influence of allosteric sites and distant protein regions on ligand binding can provide valuable insights into drug design.

The reliability and consistency of the experimental measurements in the datasets should be ensured. More precisely, there is a need to address multiple binding affinity reports for the same protein-ligand pair. Sometimes, the same protein-ligand complex may have reported different binding affinity values. The problem can arise from various factors, including experimental conditions, measurement techniques, or data curation practices. A strategy for handling multiple affinity reports must be devised to address these inconsistencies. Options include selecting a representative value, considering a range, or employing consensus methods. The rationale behind the chosen approach should be clearly documented to maintain transparency.

Duplicate entries should be appropriately addressed. Duplicate entries can introduce biases and affect the models generalizability by over-representing certain instances. Identifying and removing duplicate entries from the integrated dataset is essential to maintaining data integrity and preventing biases. This can be achieved by utilizing common identifiers, such as PDB IDs, UniProt IDs, or compound IDs, to match and eliminate redundant records.

Finally, recent studies have highlighted the efficacy of Voronoi entropy as ligand molecular descriptors. The innovative approach, as demonstrated by Sergey *et al.* [150], emphasizes the importance of integrating advanced molecular descriptors into predictive models to enhance accuracy and insight. Future research could explore the synergies between GNNs and Voronoi entropy, leveraging the unique capabilities of both approaches to improve binding affinity prediction models.

## CONCLUSION

In conclusion, this survey provides a comprehensive overview of various preprocessing steps and deep learning models for predicting the interaction strength between proteins and ligands. Additionally, our analysis of commonly used datasets offers insights into the diverse properties of these data, such as molecular weights, length of sequences, and the octanol-water partition of proteins and ligands. The state-of-the-art approach for BAP currently relies on GNN. However, despite their effectiveness, GNNs still require further efforts for interpretability and explainability. Moving forward, exploring additional avenues, such as generative AI and incorporating new molecular descriptors, is essential to enhance the predictive performance of DL models for BAP. Our work serves as a valuable starting point for both new researchers entering the field of protein-ligand binding affinity and experienced researchers. It offers guidance on dataset exploration, best practices, and effective model building while highlighting and addressing the challenges encountered.

## Figures and Tables

**Fig. (1):**
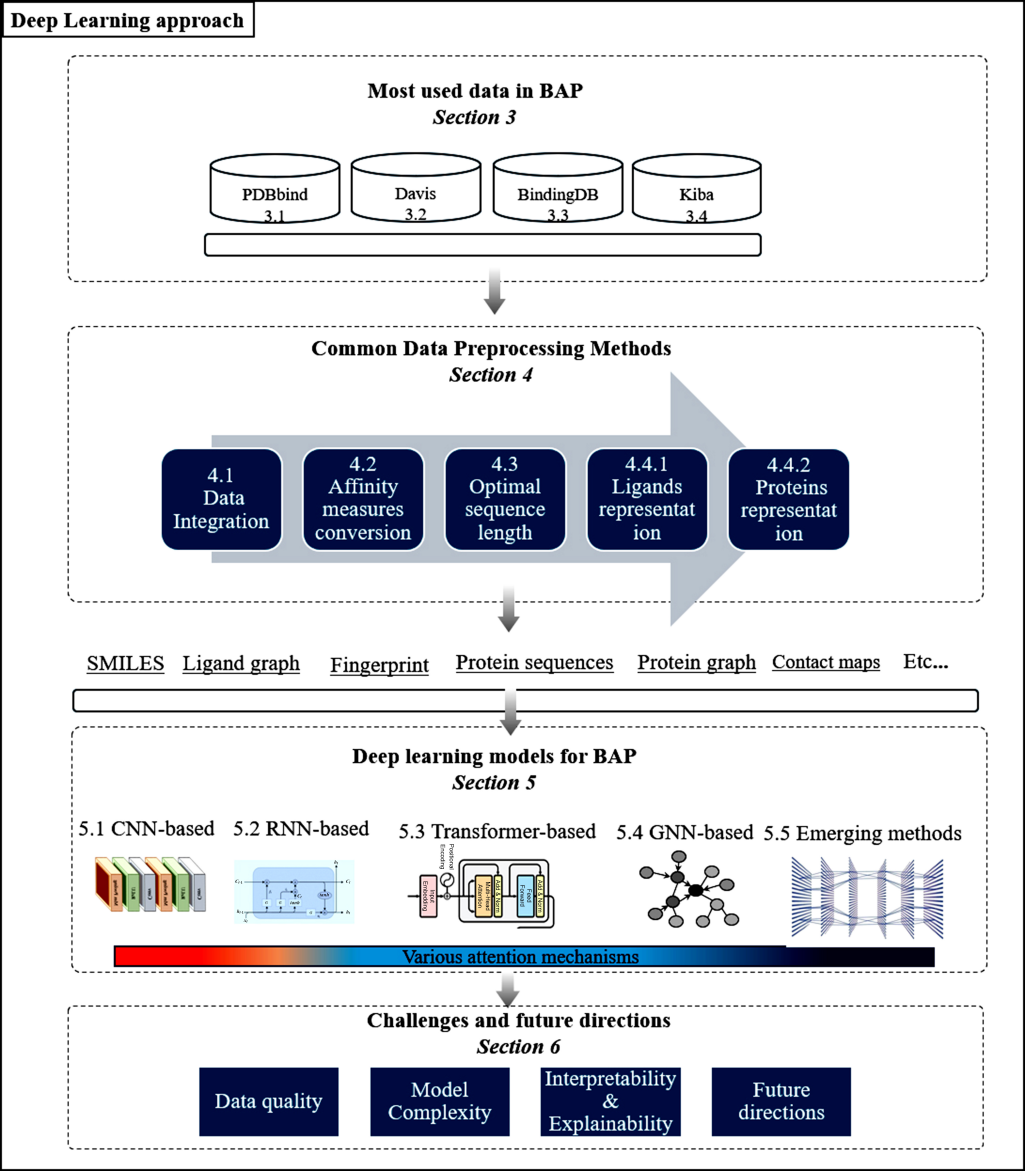
Proposed survey’s structure.

**Fig. (2):**
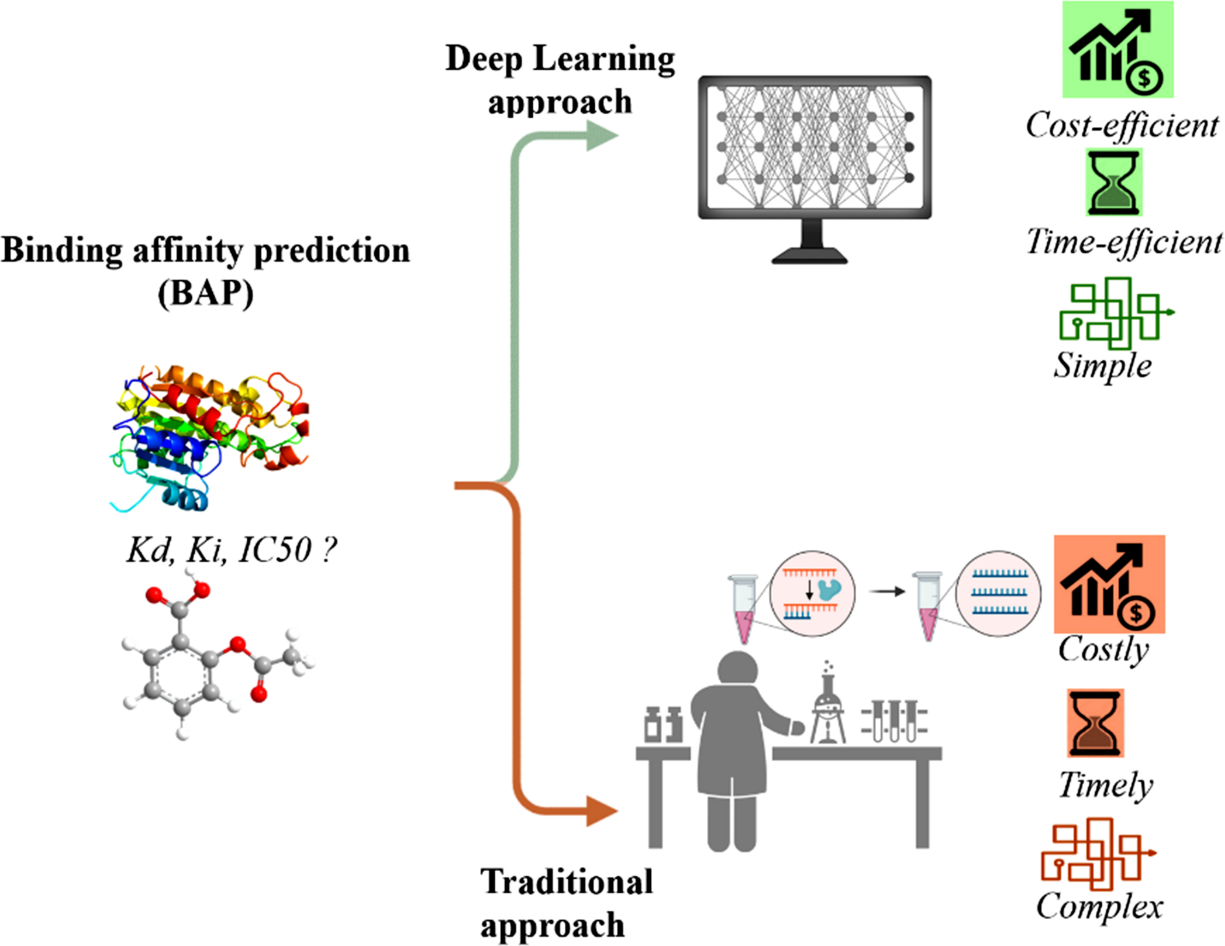
Deep learning *versus* traditional approach for BAP.

**Table 1:** Protein and ligand maximum lengths adopted by various authors.

**Models**	**Datasets**	**Protein Max Length**	**Ligand Max Length**
Electra-DTA [1]	BindingDB, Kiba, Davis	1000	100
ML_DTI [2]	Davis, Metz, Kiba	1200	100
Multi-PLI [3]	PDBbind, Davis	1200	200
GraphDTA [4]	Davis, Kiba	1000	-
WideDTA [5]	Davis	1000	85
DeepCDA [6]	Kiba, Davis, BindingDB	1000	100
ResBiGAAT [7]	PDBbind, CSAR-HiQ	1000	150
DeepDTA [8]	Davis	1200	85
	Kiba	1000	100
DeepGLSTM [9]	Davis, KIBA, DTC, Metz, ToxCast, STITCH	1000	-
Zhu *et al.* [10]	Davis, Metz, KIBA	1200	-
ColdDTA [11]	Davis KIBA	1200	
PCNN-DTA [12]	KIBA, Davis, BindingDB	1000	100
CAPLA [13]	PDBbind, CSAR-HiQ	1000	150
Zhang *et al.* [14]	Davis, Kiba	1000	100

**Table 2:** CNN-based methods for BAP.

**Models**	**Ligand Input Representation**	**Protein Input Representation**	**Ligand and Protein Feature Learning**	**Comment**
DeepDTA [8]	Lebel encoding of SMILES	Lebel encoding of AA seq	L: 1D CNN P: 1D CNN	No attention mechanism
AttentionDTA [15]	Lebel encoding of SMILES	Lebel encoding of AA seq	L: 1D CNN P: 1D CNN	Additional two-side multi-head attention mechanism
HyperAttentionDTI [91]	Lebel encoding of SMILES	Lebel encoding of AA seq	L: 1D CNN P: 1D CNN	Attention vector for each amino acid-atom pair
CAPLA [13]	one-hot encoding of SMILES	one-hot encoding of AA seq + SSE + PP	L: 1D CNN P: 1D CNN	Added protein-binding pocket AA seq the SSE
DeepDTAF [17]	Lebel encoding of SMILES	one-hot encoding of AA seq + SSE + PP	L: dilated convolution P: dilated convolution	Integration of protein-binding pocket and structural properties
WideDTA [5]	Lebel encoding of SMILES + LMCS	Lebel encoding of AA seq + Protein motifs and domains	L: 1D CNN P: 1D CNN	Combined 4 sequential features
[18]	ECFP4	PSC	L: 1D CNN P: 1D CNN	The 8420-dimensional feature vector for each protein sequence
DeepLPI [19]	Mol2Vec	ProSE	L: 1D CNN P: 1D CNN	CNN combined with LSTM
DeepCDA [6]	SMILES	Protein n-gram	L: 1D CNN P: 1D CNN	CNN combined with LSTM
FingerDTA [20]	one-hot encoding of SMILES + ECFP	one-hot encoding of AA seq + Target fingerprint	L: FC + 1D CNN P: FC + 1D CNN	Used 3 dense convolution blocks of 1D CNN
PCNN-DTA [12]	Lebel encoding of SMILES	Lebel encoding of AA seq	L: Residual 1D CNN P: Residual 1D CNN	Integration of double-sided MultiHead attention into a pyramid network
OnionNet [21]	RFEPSC	RFEPSC	L: 2D CNN P: 2D CNN	Contacts are grouped into different distance ranges.
OnionNet-2	Atom residues of complexes	Atom residues of complexes	L: 2D CNN P: 2D CNN	168 residue-atom combinations per shell
SimCNN-DTA [22]	drug similarity matrix	protein similarity matrix	L:2D CNN P:2D CNN	Similarity-based on the Tanimoto coefficient and normalized Smith-Waterman score
MDeePred [23]	ECFP4	structural + evolutionary + PP	L: FFN P: 2D CNN	1024-dimensional ECFP4 fingerprint generated from ligand SMILES
MPS2IT-DTI [24]	Molecule image	Protein image	L: 2D CNN P: 2D CNN	Treated drugs and protein sequences as 2D images
Pafnucy [25]	3D atomic coordinate of 19 channels		L: 3D CNN P: 3D CNN	The complex was cropped to a 20-Å cubic box centered on the ligands geometric midpoint.
KDEEP [26]	3D grid of 16 channels		L: 3D CNN P: 3D CNN	Van der Waals radius was employed for each atom type.
Sfcnn [27]	3D grid of 28 channels		L: 3D CNN P: 3D CNN	Atoms in protein-ligand complexes were grouped into 28 categories
DeepAtom [28]	3D grid of 24 channels		L: 3D CNN P: 3D CNN	11 Arpeggio atom types were used
AK-Score [29]	3D grid of 16 channels		L: 3D CNN P: 3D CNN	Van der Waals radius was employed for each atom type.
SE-OnionNet [30]	3D grid of 64 channels		L: 3D CNN P: 3D CNN	Eight element types were chosen to characterize ligand-protein contacts

**Abbreviations:** L: ligand; P: Proteins; ECFP: Extended-connectivity fingerprints; AA seq: Amino acid sequence; SSE: Secondary structure; PCS: protein sequence composition; RFEPSC: rotation-free element-pair-specific contacts; LMCS: ligand max common substructure; PP: physicochemical properties; ProSE: protein sequence embedding.

**Table 3:** Graph-based models for BAP.

**Models**	**Ligand Input Representation**	**Protein Input Representation**	**Ligand Feature Learning**	**Protein Feature Learning**
GraphDTA [4]	Molecular graph	one-hot encoding of AA seq	GCN\GAT\GIN\GATGCN	CNN
DeepGLSTM [9],	Molecular graph	Lebel encoding of AA seq	Multiblock GCN	Bi-LSTM
GDGRU-DTA [38],	Molecular graph	Lebel encoding of AA seq	GNN	GRU/BiGRU
EmbedDTI [60]	Atom graph and substructure graph	AA seq	GCN with attention	1D CNN
DeepGS [59]	Molecular graph + Smi2Vec	Prot2Vec	GAT + BiGRU	CNN
Dgraph-DTA [61]	Molecular graph	Protein graph	GCN	GCN
X-DPI [62]	Molecular graph + Mol2vec embedding	Protein graph + TAPE embedding	GCN	GCN
WGNN-DTA [63]	Molecular graph	Weighted protein graph	GCN/GAT	GCN/GAT
DGDTA [36]	Molecular graph	Lebel encoding of AA seq	Dynamic GAT + GCN	Bi-LSTM + CNN
PSG-BAR [64]	Molecular graph	Protein graph	Residual GAT	Residual GAT
GraphBAR [66]	Binding complexes graph	Binding complexes graph	Multiblock GCN	Multiblock GCN
APMNet [67]	Binding complexes graph	Binding complexes graph	GCN	GCN
LGN [71]	Molecular graph + IFP	Complexes graph	GIN	GNN
PLANET [72]	Molecular graph	Protein graph	GNN	EGCL
GraphscoreDTA [73]	Molecular graph	Protein graph + Interaction Graph	GNN-GRU	GNN + GNN-GRU
GraphATT-DTA [74]	Molecular graph	Lebel encoding of AA seq	GNN (GAT/GIN/GCN/MPNN/DMPNN)	1D CNN
AttentionMGT-DTA [75]	Molecular graph	Protein graph	Graph transformer	Graph transformer
GLCN-DTA [76]	Molecular graph	Protein graph	GLCN	GLCN
IEDGEDTA [77]	Molecular graph	Protein graph	Edge-GCN	1D-GCN
SAG-DTA [78]	Molecular graph	Lebel encoding of AA seq	GCN	1D CNN

**Abbreviations:** AA seq: Amino acid sequence; GNN — graph neural network; MLP — multilayer perceptron; GCN: graph convolutional network; GAT: graph attention network; GIN: graph isomorphism network; CNN: convolutional neural network; FC: fully connected layer; GRU: gated recurrent unit; EGCL: Equivariant graph convolutional layer, MPNN: message passing neural network, IFP: interaction fingerprint; GLCN: Graph learning convolutional network.

## LIST OF ABBREVIATIONS

BAP: Binding Affinity Prediction
BIGRU: Bidirectional Gated Recurrent Unit
CNN: Convolutional Neural Network
CPI: Compound-Protein Interaction
DL: Deep Learning
GNN: Graph Neural Network
GRU: Gated Recurrent Unit
IC_50_: Half Maximal Inhibitory Concentration
KD: Equilibrium Dissociation Constant
KI: Inhibition Constant
LOGP: Octanol-Water Partition
LSTM: Long Short-Term Memory
ML: Machine Learning
PLA: Protein-Ligand Binding Affinity
PLI: Protein-Ligand Interaction
RESBIGAAT: Residual Bidirectional Gated Recurrent Unit with Attention
RNN: Recurrent Neural Network
SMILES: Simplified Molecular Input Line Entry System
